# The prevalence and predictive factors of dentine hypersensitivity among adults in Turkey

**DOI:** 10.1186/s12903-023-03137-1

**Published:** 2023-07-11

**Authors:** Gunseli Katirci, Esra Uzer Celik

**Affiliations:** 1 Department of Restorative Dentistry, Faculty of Dentistry, Suleyman Demirel University, Isparta, Turkey; 2 Department of Restorative Dentistry, Faculty of Dentistry, İzmir Katip Celebi University, Izmir, Turkey

**Keywords:** Dentine hypersensitivity, Cervical lesion, Gingival recession, Thermal hypersensitivity, Evaporative hypersensitivity

## Abstract

**Aim:**

This study sought to determine the prevalence of dentine hypersensitivity (DH) among adults in Turkey. Also, to ascertain the association between DH and both etiological predictors and demographic patient characteristics.

**Material method:**

Using a questionnaire and thermal and evaporative tests, 259 women and 209 men in the age range of 18 to 72 were analyzed. Individually, a clinical evaluation of DH signs was conducted. The DMFT index, gingival index, and gingival bleeding were reported for each subject. The gingival recession and tooth wear of sensitive teeth were also evaluated. Pearson Chi-square test was used to compare categorical data. Logistic Regression Analysis was used to examine the risk factors of DH. Data with dependent categorical variables were compared using the McNemar-Browker test. The significance level was *p* < 0.05.

**Results:**

The average age of the population was 35.6 years. In the present study, a total of 12,048 teeth were analyzed. 1755 had thermal hypersensitivity (14.57%), while 470 experienced evaporative hypersensitivity (3.9%). The incisors were the teeth most impacted by DH, whereas the molars were the least affected. Exposure to cold air and sweet foods, gingival recession, and the presence of noncarious cervical lesions were all strongly linked to DH (Logistic regression analysis, *p* < 0.05). The cold stimulus increases sensitivity more than the evaporation stimulus.

**Conclusion:**

Significant risk factors for both thermal and evaporative DH include cold air, consumption of sweet food, presence of noncarious cervical lesions, and gingival recession. More epidemiological research in this area is still required to fully characterize the risk factors and implement the most effective preventive interventions.

## Introduction

Dentin hypersensitivity (DH) is an oral disorder characterized by acute discomfort of short duration emerging from exposed dentine in response to evaporative, osmotic, and tactile stimuli that cannot be accounted for by any other form of tooth disturbance or disease [1–3]. It can be described by hydrodynamic theory, and hypersensitivity lesions have a high number of dentinal tubules that have extended to the surface and are open to the pulp [4]. Exogenous heat or mechanical stimulation can produce rapid fluid movement in the narrow dentine tubules, activating the nerve terminals within the pulp and dentine, hence causing DH [1, 5].

DH is a significant issue that can influence patients' quality of life [6, 7]. Tooth wear, primarily in the form of erosion in occlusal or facial/buccal or lingual/palatal surfaces, gingival recession, age, gender (female), education level, and occupation or socioeconomic status have been implicated as factors of DH [8–13]. Other contributing factors include drinking habits, oral hygiene practices such as the abrasiveness of toothpaste, and toothbrushes, flament stiffness, toothbrushing procedures, frequency, and toothbrushing pressure. Smoking is an additional risk factor. It was stated that smoking is a significant risk factor for periodontal disease and that exposure to root surfaces and gingival recession resulting in dentin hypersensitivity is a widespread effect of periodontal disease [13–17].

It has been found that DH is most common between the ages of 30 and 40, ranging from 3 to 98% [3, 18]. This extensive portfolio is due in part to patient selection criteria, heterogeneity in diagnostic methodologies, and time gaps between investigations [18]. The multiple causes of DH should be investigated and determined. It is essential to identify the causes of DH, as treatment should involve the minimization and elimination of hypersensitive triggers [19]. The literature presents a variety of diagnostic criteria employed to identify DH [7]. Certain studies utilize self-administered questionnaires to inquire about tooth sensitivity, while others rely on professional clinical examination methods for the identification of DH [20]. Individuals often report tooth sensitivity, which may not only be a result of DH but also other prevalent oral diseases such as caries or periodontal issues, thereby leading to a higher prevalence. The researches available can be broadly categorized into two groups based on the diagnostic approach: self-questionnaires and clinical records. Despite these methods, the prediction interval remains considerably wide, ranging from 13–57% or 4–74%, respectively. Several factors have been proposed to account for these variations, including sample characteristics such as ethnicity, workplace, periodontal status, dental care regimen, oral hygiene habits, and socio-economic conditions [13, 20]. The determination of criteria for DH may hinge on two contrasting approaches: a passive approach that relies on the subject's reported pain experience, and an active approach that involves various mechanical and thermal stimulations [20, 21]. An additional complicating factor is the episodic nature of the condition, which can either provoke or alleviate pain symptoms [20].

It was stated that the results of the prevalence and risk variables overlapped in the studies, making it necessary to conduct additional epidemiologic research in order to accurately identify the risk factors and apply appropriate preventative methods. In several prevalence studies conducted on the Turkish population, sensitivity was shown to range from 5.1% to 51.6% [1, 15, 21, 22]. All of these investigations, however, involved participants who resided in areas that were dissimilar to those of our research. It was judged vital to investigate the probable factors driving dentin sensitivity utilizing epidemiological research in order to develop an appropriate therapy for DH in particular groups [17, 23]. This study sought to determine the prevalence of DH among adults who were referred to the restorative dentistry clinic at a university in Turkey. Also, to ascertain the association between DH, etiological predictors, and demographic patient characteristics.

## Methods

DH was assessed in this cross-sectional, single-center investigation in adult patients. This study was conducted in the clinics of the Restorative Dentistry Department at the Faculty of Dentistry, Suleyman Demirel University. The Ethics Committee of Suleyman Demirel University Faculty of Medicine granted approval for the current research with protocol 2020/25/361. The investigation was conducted between January and June of 2021.

### Sample selection

The sample size was calculated using a power analysis performed in G-Power (G*Power Ver. 3.0.10, Franz Faul, Universität Kiel, Germany). It was used with 95% confidence (1- α) and 95% power (1-β). The prevalence in the literature was 19% [3]. It was calculated that at least 457 patients were to be evaluated. 11 subjects were added to avoid potential data loss and the study involved 468 participants.

Participants in the study were presumed to be in good general health and willing to take part in the examination. Individuals who were taking analgesic medications, required antibiotics for dental treatment, or had recently undergone oral local anaesthetic within the previous 24 h were excluded. The current study did not include teeth with fluorotic stains, endodontic treatment, orthodontic appliances, prosthetic crowns, restorations, dental cavities, fractures, or anything else that would mask the sensitivity symptoms.

### Assessments

The study employed a two-step research process, encompassing both a questionnaire and a clinical examination. A self-administered questionnaire, modeled after those used in previous studies to identify risk factors for DH, was prepared [3, 7, 24]. The examiner read the questions aloud and recorded all patient information directly onto the questionnaire.

The questionnaire was designed to gather demographic data such as age and gender, as well as information on oral hygiene routines, including brushing frequency (less than once daily, once daily, twice daily, thrice daily), handedness (right or left-handed), and the presence of excessive pressure during brushing. This data was collected from the entire patient population.

The questionnaire also included queries related to self-reported DH, such as the frequency of tooth hypersensitivity (never, often, occasionally, rarely) and the stimuli that triggered DH (cold air, sweet food, cold drink/ice, etc.). Additional questions addressed habits that could potentially affect oral health, including the frequency of bother from the view of teeth (never, often, occasionally, rarely), snoring (never, often, occasionally, rarely), and chewing gum (never, often, occasionally, rarely).

The questionnaire also evaluated the consumption of acidic components in the diet, specifically the frequency of consumption of soft drinks (cola, fanta, etc.) and alcoholic beverages (never, often, occasionally, rarely). Furthermore, it gathered information on parafunctional behaviors (such as bruxism) and dental visitation patterns, specifically the time interval between the last and current dental visit, from the entire patient population.

Each participant underwent a clinical examination to evaluate the signs of dentine hypersensitivity. To ensure consistency and eliminate potential discrepancies due to inter-examiner variability, all clinical examinations were conducted by a single, trained dental investigator. This investigator received specialized training in the diagnosis and management of DH, adhering to the methodology recommended by the Canadian Advisory Board on Dentine Hypersensitivity [25]. To validate the reliability of the method, the investigator examined ten university students, who were not part of the study. This resulted in an intra-agreement rate of 98%, indicating the suitable reproducibility of the method for evaluating DH [12, 26].

The evaluations did not take into account third molars. In accordance with the WHO standards for epidemiological studies, decayed, missing, and filled teeth (DMFT) indices were employed to assess the state of dental caries [27]. The gingival inflammation was shown using Loe and Silness' Gingival Index (0 ± 3) [28]. The evaluation of gingival recession and the presence or absence of gingival bleeding were conducted using a 1-mm graduated periodontal probe, specifically a Williams periodontal probe. The extent of the gingival recession was gauged at the midpoint of the vestibular surface, spanning from the enamel-cementum junction to the free gingival margin, and was documented in millimeters [2]. Noncarious cervical lesions were divided into concave and wedge-shaped morphologies. The Basic Erosive Wear Examination (BEWE) was used to evaluate the wear on the buccal and palatal/lingual teeth. 0 indicates no erosive wear, 1 indicates early tooth loss, 2 indicates surface loss of less than 50%, and 3 indicates wear with tissue loss of more than 50% of the surface in addition to the location of the lesion [29].

Each tooth that had fully erupted in the month prior was examined for DH using a thermal test and cold air was applied to the cervical region of the buccal side. An iced stick was placed on the vestibular surface of each tooth to conduct the cold test. The stimulus was kept going for a maximum of 5 s until the patient's painful reaction [2]. Ten minutes later, the evaporative stimulus generated by an air–water syringe was utilized to measure the degree of tooth sensitivity. The air jet was aimed perpendicularly at the buccal surface of the tooth for the duration of the two seconds, around 1 cm away [30]. A polyester band was placed over the adjacent teeth to prevent false-positive findings. The pain was classified using the 1994 Schiff ordinal scale (0 = Participant does not respond to sensitivity, 1 = Participant responds to stimulus but does not request discontinuation of stimulus, 2 = Participant responds to stimulus and requests discontinuation or moves away from a stimulus, 3 = Participant responds to air stimulus, considers stimulus to be painful, and requests discontinuation of the stimulus) [31].

### Statical analysis

SPSS was used for statistical analysis (Statistical Package for the Social Sciences, version 23.0, IBM Corp., Armonk, NY). The Kolmogorov–Smirnov test was used to determine the data's normality. The frequency of categorical variables was determined using descriptive statistical analyses. Moreover, mean, standard deviation and median (minimum and maximum) values were employed to produce quantitative variables for descriptive statistical analyses. Categorical data were compared using the Pearson Chi-square test. The independent variables were included in the multivariate model using the Backward technique, and Logistic Regression Analysis was utilized to analyze the risk factors of DH. The McNemar Browker Test was used to compare dependent categorical data. The level of significance was set at *p* < 0.050.

## Results

The present study comprised 259 women and 209 males in the age range of 18–72 years old. The population's average age was 35.6. In the current study, 157 subjects in total self-reported experiencing dentine hypersensitivity (Table 1). With regard to the patients, 251 had thermal DH (55%) and 97 had evaporative DH (22%) as determined by a clinical assessment. In individuals who did not complain of sensitivity, the rate of thermal sensitivity was 41%, and the rate of evaporative sensitivity was 14% (Table 2). In this study, 12,048 teeth in total were evaluated 1755 of them (14.57%) experienced thermal hypersensitivity, while 470 (3.90%) had evaporative hypersensitivity. The incisors and molars from the DH were the most and least affected, respectively (Table 3).

**Table 1:** Patients' demographics (*n* = 468)

Variables	Mean	SD
**Age**	35.60	13.13
**Gender**	**n**	**%**
Male	209	44.7
Female	259	55.3
**DH self-report (*n* = 468)**	**n**	**%**
Yes	157	32.8
No	311	67.2

**Table 2:** Association between self-reported and clinically diagnosed DH (*n* = 468)

	**Self-reported**				**Test Statistic**	**p**
	**Never n(%)**	**Often n(%)**	**Sometimes n(%)**	**Rarely n(%)**		
**Thermal sensitivity**						
Absent	178 (58.7)	6 (15.8)	8 (9)	11 (50)	–-	–-
Present	125 (41.3)	32 (84.2)	81 (91)	11 (50)		
**Evaporative sensitivity**						
Absent	253 (85.8)	15 (44.1)	56 (64.4)	17 (81)	–-	–-
Present	42 (14.2)	19 (55.9)	31 (35.6)	4 (19)		

McNemar Browker Test

**Table 3:** Sample size for various DH tests

**Thermal Test** (*n* = 1755, 14.57%)		
**Teeth**	**n**	**%**
Incisives	1194	68.03
Canines	295	16.81
Premolars	223	12.71
Molars	43	2.45
**Evaporative Test** (*n* = 470, 3.90%)		
**Teeth**	**n**	**%**
Incisives	277	58.94
Canines	91	19.36
Premolars	89	18.94
Molars	13	2.77

There was a statistically significant disparity between the thermal and evaporative sensitivity of noncarious cervical lesions. Noncarious cervical lesions had a prevalence of thermal and evaporative sensitivity of 91% and 45%, respectively (Pearson Ki-Kare Test, *p* = 0.005, *p* < 0.001; Table 4).

**Table 4:** The distribution of thermal and evaporative sensitivity in relation to the existence of noncarious cervical lesions of teeth (*n* = 468)

	**Noncarious cervical lesion**		**Test Statistic**	p
	**Absent**	**Present**		
**Thermal sensitivity**				
Absent	230 (14.9)	36 (9.4)	7.952	**0.005**
Present	1311 (85.1)	348 (90.6)		
**Evaporative sensitivity**				
Absent	1034 (67.1)	210 (54.7)	20.714	** < 0.001**
Present	507 (32.9)	174 (45.3)		

Pearson Chi-Square Test, *p* < 0.05

Thermal and evaporative sensitivity risk variables were analyzed using univariate and multivariate logistic regression (Table 5, 6 and 7). Age, self-reported sensitivity, cold air, sweet and cold nutrition, bother from the view of teeth, and use of soft and alcoholic drinks were significantly associated with thermal hypersensitivity, as shown by univariate logistic regression analysis. The risk of thermal sensitivity was observed to decrease by 0.985 times with age (OR = 0.985, %95 Cl = 0.971–0.999, *p* = 0.041; Table 5). Thermal sensitivity increased with self-reported sensitivity (OR = 0.985, %95 Cl = 0.971–0.999, *p* = 0.041; Table 5), cold nutrients (OR = 4.727, %95 Cl = 1.582–14.129, *p* = 0.005; Table 6), bother from the view of teeth (OR = 2.136, %95 Cl = 1.329–3.433, *p* = 0.002; Table 6), and consuming soft drinks sometimes (OR = 0.035%95 Cl = 0.002–0.546, *p* = 0.017; Table 6). The thermal sensitivity of patients with the habit of consuming alcoholic drinks sometimes was 0.2226 times lower than that of patients without this behavior (OR = 0.226, %95 Cl = 0.061–0.836, *p* = 0.026; Table 6). Using the Backward Wald approach, the independent variables were included in the multivariate model. In the multivariable model, thermal sensitivity increased 4.677 times when bruxism was present (OR = 4.677, %95 Cl = 1.45–15.08, *p* = 0.010; Table 7).

**Table 5:** Prevalence of dentin hypersensitivity as determined by thermal and evaporative stimulation in adults (*n* = 468)

Varıables	Thermal stımulus						Evaporatıve stımulus					
	**Absent n (%)**	**Present n (%)**	**Univariate**		**Multivariate**		**Absent**	**Present**	**Univariate**		**Multivariate**	
			**OR (95%CI)**	**p**	**OR (95%CI)**	**p**			**OR (95%CI)**	**p**	**OR (95%CI)**	**p**
**Age**	–-	–-	0.985 (0.971–0.999)	**0.041**	–-	–-	–-	–-	1.01 (0.993 – 1.027)	0.251	1.062 (1.024 – 1.102	**0.001**
**Cender**												
Male	93 (46.3)	108 (53.7)	1.099 (0.758–1.594)	0.617	–-	–-	163 (83.6)	32 (16.4)	1.829 (1.14–2.936)	**0.012**	–-	**––**
Female	112 (43.9)	143 (56.1)					181 (73.6)	65 (26.4)				
**Brushing frequency**												
< 1 daily	58 (45.7)	69 (54.3)					97 (77)	29 (23)			–-	
1/day	49 (39.8)	74 (60.2)	1.269 (0.768–2.098)	0.352	––	––	86 (73.5)	31 (26.5)	1.206 (0.673–2.161)	0.530	–-	**–-**
2/day	87 (46)	102 (54)	0.986 (0.628–1.548)	0.949			146 (80.2)	36 (19.8)	0.825 (0.475–1.433)	0.494	–-	**–-**
3/day	11 (64.7)	6 (35.3)	0.458 (0.16–1.316)	0.147			15 (93.8)	1 (6.3)	0.223 (0.028–1.761)	0.155	–-	**–-**
**Right or left-handed**												
Right-handed	189 (45.3)	16 (41)	1.192 (0.612–2.321)	0.606	––	–-	319 (79.2)	84 (20.8)	1.975 (0.969–4.025)	0.061	–-	**–-**
Left-handed	16 (41)	23 (59)					25 (65.8)	13 (34.2)				
**Excessive pressure during brushing**												
Absent	162 (47.2)	181 (52.8)	1.415 (0.915–2.191)	0.119	–-	–-	268 (79.5)	69 (20.5)	1.398 (0.837–2.336)	0.201	–-	**–-**
Present	43 (38.7)	68 (61.3)					75 (73.5)	27 (26.5)				
**Self-reported hypersensitivity**												
Never	178 (58.7)	125 (41.3)					253 (85.8)	42 (14.2)				
Often	6 (15.8)	32 (84.2)	7.595 (3.083–18.707)	** < 0.001**	4.947 (0.72–33.983)	0.104	15 (44.1)	19 (55.9)	7.63 (3.598–16.179)	** < 0.001**	–-	–-
Occasionally	8 (9)	81 (91)	14.418 (6.733–0.877)	** < 0.001**	11.355 (1.734–4.362)	**0.011**	56 (64.4)	31 (35.6)	3.335 (1.93–5.762)	** < 0.001**	–-	–-
Rarely	11 (50)	11 (50)	1.424 (0.599–3.387)	0.424	0.716 (0.103–4.971)	0.735	17 (81)	4 (19)	1.417 (0.455–4.419)	0.548	–-	–-
**Cold air**												
Never	19 (26)	54 (74)					53 (75.7)	17 (24.3)				
Often	3 (16.7)	15 (83.3)	1.759 (0.458–6.755)	0.411	–-	–-	6 (37.5)	10 (62.5)	5.196 (1.645–16.413)	**0.005**	0.812 (0.145–4.561)	0.813
Occasionally	4 (7.8)	47 (92.2)	4.134 (1.313–13.017)	**0.015**	–-	–-	25 (50)	25 (50)	3.118 (1.432–6.79)	**0.004**	2.993 (0.419–21.38)	0.274
Rarely	4 (26.7)	11 (73.3)	0.968 (0.275–3.405)	0.959	–-	–-	12 (85.7)	2 (14.3)	0.52 (0.106–2.557)	0.421	2.082 (0.364–11.897)	0.410
**Sweet food**												
Never	16 (19)	68 (81)					54 (67.5)	26 (32.5)				
Often	1 (7.1)	13 (92.9)	3.059 (0.372–25.119)	0.298	–-	–-	7 (53.8)	6 (46.2)	1.78 (0.543–5.832)	0.341	3.083 (0.61–15.576)	0.173
Occasionally	4 (10)	36 (90)	2.118 (0.659–6.808)	0.208	–-	–-	19 (48.7)	20 (51.3)	2.186 (0.999–4.784)	0.050	9.24 (1.239–68.906)	**0.030**
Rarely	9 (47.4)	10 (52.6)	0.261 (0.091–0.749)	**0.012**	–-	–-	16 (88.9)	2 (11.1)	0.26 (0.056–1.214)	0.087	6.566 (1.22–35.351)	**0.028**

Logistic regression analysis, *OR* Odds ratio, *Cl* Confidence interval, the bold number indicates statistical significance, *p* < 0.05

**Table 6:** Prevalence of dentin hypersensitivity as determined by thermal and evaporative stimulation in adults (*n* = 468)

Varıables	Thermal stımulus						Evaporatıve stımulus					
	**Absent**	**Present**	**Univariate**		**Multivariate**		**Absent**	**Present**	**Univariate**		**Multivariate**	
			**OR (95%CI)**	**p**	**OR (95%CI)**	**p**			**OR (95%CI)**	**p**	**OR (95%CI)**	**p**
**Cold (drinks, ice..)**												
Never	8 (42.1)	11 (57.9)					13 (68.4)	6 (31.6)				
Often	5 (15.2)	28 (84.8)	4.073 (1.091–15.203)	0.037	––	–-	18 (58.1)	13 (41.9)	1.565 (0.47–5.205)	0.465		
Occasionally	12 (13.3)	78 (86.7)	4.727 (1.582–14.129)	**0.005**	––	–-	51 (59.3)	35 (40.7)	1.487 (0.516–4.286)	0.463	–-	–-
Rarely	5 (33.3)	10 (66.7)	1.455 (0.356–5.945)	0.602	––	–-	14 (100)	0 (0)	–-	–-	–-	–-
**Bother from the view of teeth**												
Never	134 (50.6)	131 (49.4)					210 (82)	46 (18)				
Often	30 (40.5)	44 (59.5)	1.5 (0.889–2.531)	0.128	–-	–-	49 (67.1)	24 (32.9)	2.236 (1.248–4.007)	**0.007**	–-	–-
Occasionally	34 (32.4)	71 (67.6)	2.136 (1.329–3.433)	**0.002**	–-	–-	73 (73)	2	1.689 (0.979–2.911)	0.059	–-	–-
Rarely	7 (58.3)	5 (41.7)	0.731 (0.226–2.36)	0.600	–-	–-	12 (100)	0 (0)	–-	–-	–-	–-
**Snoring**												
Never	145 (45.6)	173 (54.4)					241 (78.8)	65 (21.2)				
Often	16 (42.1)	22 (57.9)	1.152 (0.583–2.276)	0.683	–-	–-	29 (76.3)	9 (23.7)	1.151 (0.519–2.552)	0.730	–-	–-
Occasionally	37 (45.1)	45 (54.9)	1.019 (0.626–1.66)	0.939	–-	–-	58 (74.4)	20 (25.6)	1.279 (0.718–2.277)	0.404	–-	–-
Rarely	7 (38.9)	11 (61.1)	1.317 (0.498–3.485)	0.579	–-	–-	16 (84.2)	3 (15.8)	0.695 (0.197–2.459)	0.573	–-	–-
**Chewing gum**												
Never	81 (47.1)	91 (52.9)					128 (76.6)	39 (23.4)				
Often	13 (50)	13 (50)	0.89 (0.39–2.031)	0.782	–-	–-	21 (80.8)	5 (19.2)	0.781 (0.276–2.209)	0.642	–-	–-
Occasionally	70 (43.2)	92 (56.8)	1.17 (0.76–1.801)	0.476	–-	–-	124 (77.5)	36 (22.5)	0.953 (0.569–1.596)	0.854	–-	––
Rarely	41 (42.7)	55 (57.3)	1.194 (0.722–1.975)	0.490	–-	–-	71 (80.7)	17 (19.3)	0.786 (0.415–1.489)	0.460	–-	–-
**Soft drinks (Cola, Fanta, etc.…)**												
Never	76 (50.7)	74 (49.3)					114 (78.1)	32 (21.9)				
Often	20 (51.3)	19 (48.7)	0.976 (0.482–1.974)	0.945	0.035 (0.002–0.546)	**0.017**	29 (76.3)	9 (23.7)	1.106 (0.475 – 2.572)	0.816	–-	–-
Occasionally	58 (37.4)	97 (62.6)	1.718 (1.088–2.711)	0.020	0.713 (0.176–2.881)	0.634	117 (77.5)	34 (22.5)	1.035 (0.599 – 1.79)	0.901	–-	–-
Rarely	51 (45.5)	61 (54.5)	1.228 (0.752 -2.006)	0.411	0.893 (0.205–3.885)	0.880	84 (79.2)	22 (20.8)	0.933 (0.506 – 1.72)	0.824	–-	–-
**Alcoholic drinks**												
Never	147 (43)	195 (57)					249 (75.2)	82 (24.8)				
Often	10 (76.9)	3 (23.1)	0.226 (0.061–0.836)	**0.026**	–-	–-	12 (100)	0 (0)	–-			
Occasionally	22 (43.1)	29 (56.9)	0.994 (0.549–1.8)	0.983	–-	–-	40 (78.4)	11 (21.6)	0.835 (0.41–1.703)	0.620	–-	–-
Rarely	26 (52)	24 (48)	0.696 (0.384–1.261)	0.232	–-	–-	43 (91.5)	4 (8.5)	0.282 (0.098–0.811)	**0.019**	–-	–-

Logistic regression analysis, *OR* Odds ratio, *Cl* Confidence interval, the bold number indicates statistical significance, *p* < 0.05

**Table 7:** Prevalence of dentin hypersensitivity as determined by thermal and evaporative stimulation in adults (*n* = 468)

Variables	Thermal stimulus						Evaporative stimulus					
	**Absent n (%)**	**Present n (%)**	**Univariate**		**Multivariate**		**Absent n (%)**	**Present n (%)**	**Univariate**		**Multivariate**	
			**OR (95%CI)**	**p**	**OR (95%CI)**	**p**			**OR (95%CI)**	**p**	**OR (95%CI)**	**p**
**Bruxism**												
Absent	144 (49.5)	147 (50.5)	0.696 (0.384–1.261)	0.232	4.677 (1.45–15.08)	**0.010**	223 (79.1)	59 (20.9)	1.187 (0.746–1.888)	0.469	–-	–-
Present	61 (37)	104 (63)					121 (76.1)	38 (23.9)				
**The time interval between the last and current dental visit**												
< 6 months	154 (43.5)	200 (56.5)	0.818 (0.401–1.671)	0.582	–-	**–-**	259 (75.7)	83 (24.3)	0.557 (0.208–1.489)	0.244	–-	–-
6–12 months	16 (48.5)	17 (51.5)	0.55 (0.238–1.272)	0.162	–-	**–-**	28 (84.8)	5 (15.2)	0,312 (0,071–1.363)	0.122	–-	–-
1–2 year	14 (58.3)	10 (41.7)	0.924 (0.389–2.195)	0.858	–-	**–-**	20 (90.9)	2 (9.1)	0,52 (0.149–1.81)	0.304	–-	–-
2–5 years	10 (45.5)	12 (54.5)	0.513 (0.142–1.851)	0.308	–-	**–-**	18 (85.7)	3 (14.3)	0,78 (0.162–3.746)	0.756	–-	–-
> 5 years	6 (60)	4 (40)	1.232 (0.395–3.84)	0.719	–-	**–-**	8 (80)	2 (20)	0,567 (0.123–2.612)	0.467	–-	–-
Not know	5 (38.5)	8 (61.5)					11 (84.6)	2 (15.4)				

Logistic regression analysis, *OR* Odds ratio, *Cl* Confidence interval, the bold number indicates statistical significance, *p* < 0.05

Analysis of univariate logistic regression revealed that gender, age, self-reported sensitivity, cold air, cold nutrition, bother from the view of teeth, and alcohol use were significantly associated with evaporative hypersensitivity. Evaporative hypersensitivity increased with self-reported sensitivity (1.062, %95 Cl = 1.024–1.102, *p* = 0.001; Table 5), cold air (OR = 5.196, %95 Cl = 1.645–16.413, *p* = 0.005; Table 5), and bother from the view of teeth (OR = 2.236, %95 Cl = 1.248–4.007, *p* = 0.007; Table 6). Rare alcohol use was associated with a 0.282-fold reduction in evaporative hypersensitivity (OR = 0.226, %95 Cl = 0.061–0.836, *p* = 0.026; Table 6). Age and sweet diet were significantly associated with evaporative hypersensitivity, according to multivariate logistic regression analysis (Table 5). Infrequent (sometimes and rarely) eating of sweet foods enhanced evaporative hypersensitivity (OR = 9.24, %95 Cl = 1.239–68.906, *p* = 0.030). In the current analysis, the probability of evaporative hypersensitivity rose with age (OR = 1.062, %95 Cl = 1.024 –1.102, *p* = 0.001; Table 5).

Thermal and evaporative sensitivity risk variables were analyzed using univariate and multivariate logistic regression (Table 8). Gingival bleeding, gingival recession, DMFT, dental plaque, and Gingival Index (GI) were significantly associated with thermal hypersensitivity. Thermal hypersensitivity increased with gingival bleeding (OR = 42.1, %95 Cl = 0–28.8, *p* = 0.005), gingival recession (OR = 86.5, %95 Cl = 0–17.1, *p* = 0.015), and noncarious cervical lesions (OR = 91.7, %95 Cl = 8.3–0, *p* = 0.005; Table 8). Thermal hypersensitivity reduced with missing teeth index (MT) (OR = 0.859, %95 Cl = 0,828–0.891, *p* < 0.001), DMFT (OR = 0.974, 95% CI: 0.951–0.997, *p* = 0.028), and GI (OR = 0.728, %95 Cl = 0.619–0.857, *p* < 0.001; Table 8). Noncarious cervical lesions were associated with an increased risk of evaporative sensitivity (OR = 1.69,%95 Cl = 1.346–2.121, *p* < 0.001; Table 8). The incidence of evaporative sensitivity was 0.167 times lower in attrition lesions compared to erosion lesions (OR = 0.167, %95 Cl = 0.042–0.666, *p* = 0.011). Sensitivity to evaporation increased with filling tooth index (OR = 1.139, %95 Cl = 1.11–1.169, *p* < 0.001). The presence of tooth plaque lowered evaporative sensitivity (OR = 0.766, 95% Cl = 0.599–0.949, *p* = 0.034; Table 8). Evaporative sensitivity increased with gingival recession, according to multivariate logistic regression analysis (OR = 120.737,%95 Cl = 4,45–3275,143, *p* = 0,004; Table 8). When the number of missing teeth increased, the risk of experiencing evaporative sensitivity reduced by a factor of 0.162% (OR = 0,162, %95 Cl = 0.046–0.562, *p* = 0.004; Table 8).

**Table 8:** Prevalence of dentin hypersensitivity as determined by thermal and evaporative stimulation in teeth

Varıables	Thermal stımulus						Evaporatıve stımulus					
	**Absent n (%)**	**Present n (%)**	**Univariate**		**Multivariate**		**Absent n (%)**	**Present n (%)**	**Univariate**		**Multivariate**	
			**OR (95%CI)**	**p**	**OR (95%CI)**	**p**			**OR (95%CI)**	**p**	**OR (95%CI)**	**p**
**Sensitivity like syndrome**												
Absent	244 (14.2)	1475 (85.8)	99.4 (0.6—0)	0.136	–-	–-	1120 (65.2)	599 (34.8)	1.312 (0.98 – 1.756)	0.068	–-	–-
Present	22 (10.4)	189 (89.6)					124 (58.8)	87 (41.2)				
**Gingival Bleeding**												
Absent	211 (15.1)	1182 (84.9)	42.1 (0 – 28.8)	**0.005**	–-	–-	890 (63.9)	503 (36.1)	0.915 (0.742 – 1.128)	0.404	–-	–-
Present	55 (10.2)	482 (89.8)					354 (65.9)	183 (34.1)				
**Gingival Recession**												
Absent	203 (12.9)	1374 (87.1)	86.5 (0 – 17.1)	**0.015**			1027 (65.1)	550 (34.9)	1.17 (0.922—1.485)	0.195	120.737 (4.451–3275.143)	**0.004**
Present	63 (17.8)	290 (82.2)					217 (61.5)	136 (38.5)				
**Noncarious cervical lesions**												
Absent	230 (14.9)	1311 (85.1)	91.7 (8.3—0)	**0.005**			1034 (67.1)	507 (32.9)	1.69 (1.346 – 2.121)	** < 0.001**	–-	–-
Present	36 (9.4)	348 (90.6)					210 (54.7)	174 (45.3)				
**Lesion type**												
Erosion	6 (16.7)	30 (83.3)					18 (50)	18 (50)				
Abrasion	18 (16.7)	90 (83.3)	1 (0.363 – 2.751)	1.000	–-	–-	69 (63.9)	39 (36,1)	0.565 (0.264 – 1.211)	0.142	–-	–-
Atrision	3 (14.3)	18 (85.7)	1.2 (0.267 – 5.4)	0.812	–-	–-	18 (85.7)	3 (14.3)	0.167 (0.042 – 0.666)	**0.011**	–-	–-
**Varıables**	**Thermal stımulus**						**EVAPORATIVE STIMULUS**					
	**Absent Mean (Sd)**	**Present Mean (Sd)**	**Univariate**		**Multivariate**		**Absent Mean (Sd)**	**Present Mean (Sd)**	**Univariate**		**Multivariate**	
			**OR (95%CI)**	**p**	**OR (95%CI)**	**p**			**OR (95%CI)**	**p**	**OR (95%CI)**	**p**
DT	1.91 (2.94)	2,16 (2.33)	1.049 (0.989—1.113)	0,110	–-		2.18 (2.54)	2.04 (2.21)	0.975 (0.938 – 1.015)	0.217	–-	**–-**
MT	3.29 (4.99)	1.44 (2.38)	0.859 (0,828 – 0.891)	** < 0.001**	–		1.64 (3.01)	1.78 (2.84)	1.016 (0.985 – 1.048)	0.324	0.162 (0.046 – 0.562)	**0.004**
FT	5.65 (4.02)	6.22 (3.75)	1.042 (1.006 – 1.08)	**0.022**	–-		5.49 (3.5)	7.33 (4.01)	1.139 (1.11 – 1.169)	** < 0.001**	–-	**–-**
**DMF**	10.62 (6.42)	9.84 (5.16)	0.974 (0.951 – 0.997)	**0.028**	–-		9.27 (5.16)	11.18 (5.49)	1.069 (1.05 – 1.088)	** < 0.001**	1.937 (1.196 – 3.137)	**0.007**
**Dental Plaque**	1.18 (0.42)	0.18 (0.39)	1.002 (0.72 – 1.394)	0.991	–-		0.2 (0.41)	0.16 (0.37)	0.766 (0.599 – 0.98)	**0.034**	98.208 (1.067—9038.633)	**0.047**
**BWE Score**												
Facial	1.00 (0)	1.17 (0,38)	–-	**–-**	–-		1.09 (0.29)	1.22 ( 0.42)	2.786 (0.707 – 10.983)	0.143	–-	**–-**
Occlussal	1.06 (0.25)	1.03 (0.16)	0.417 (0.035 – 4.897)	0.486	**–-**		1.05 (0.22)	1 (0**)**	**–-**	–-	**–-**	**–-**
**Gingival Index**	1.20 (0.83)	0.99 (0.79)	0.728 (0.619 – 0.857)	** < 0.001**	**–-**		1.03 (0.81**)**	1.01 (0.79)	0.977 (0.869 – 1.098)	0.696	5.324 (0.91—31.142)	0.064

Logistic regression analysis, *OR* Odds ratio, *Cl* Confidence interval, the bold number indicates statistical significance, *p* < 0.05

## Discussion

In the present study, self-reported thermal hypersensitivity prevalence was 84.2% and evaporative sensitivity prevalence was 55.9%. The clinical examination of the teeth revealed a prevalence of 14.57% for thermal sensitivity and 3.9% for evaporative sensitivity. There was a significant difference between self-reported sensitivity and clinically obtained DH in our investigation, as there was in prior studies [13, 23, 32–34]. The range of the self-reported DH was 25% to 49.7%. Moreover, the clinical DH rate varied from 4.1% to 34.5%. In contrast to our findings, Demirci et al. discovered that in the Turkish community, the rate of DH with clinical observation was greater than the self-reported sensitivity [1]. The self-reported sensitivity may be overstated in comparison to clinical observation in the current investigation. Consistently with this finding, the reasons for this difference were explained by some factors such as the overall mean pain score being in the light range, implying that pain had a great impact on the patient’s everyday life. In addition, unlike the current study, Demirci et al. used tactile and evaporative tests to evaluate DH in their study. Using different stimulation as thermal and evaporative tests could lead to sensations and pain symptoms, so started self-reported DH for the patients in the present study [1].

In line with most of the previous research, the present study discovered that females had the highest frequency of DH. According to several studies, females have more sensitive teeth than males [25, 35]; however, the cause of the gender disparity in the studies was not well reported. Yet, it was linked to the female's effective plaque management [10, 36]. Ameresana et al. observed that women had a high tendency to over-report sensitivity to their underlying medical illnesses. In addition, females were more motivated to receive treatment for DH than males [36].

Which teeth are most susceptible to dentin sensitivity is a topic of debate in the scientific literature. According to several research, an adult's premolars were the teeth most commonly impacted by DH. Premolars' position in the dental arc, which makes them more susceptible to excessive brushing power, gingival recession, and the development of noncarious cervical lesions, were all cited as contributing factors to the development of DH [37–39]. According to Barosso et al., premolars and incisors were the teeth most commonly impacted by DH [40]. In other investigations, incisors and canines were the most impacted teeth since they have thinner enamel than the other teeth, which matched our findings [3, 19, 41–46].

Many investigations indicated a substantial relationship between noncarious cervical lesions and DH, which corresponded with our results [1, 11, 32, 47]. In our research, erosion lesions had a greater probability of evaporative sensitivity compared to attrition lesions. According to Teixeira et al., wedge-shaped noncarious cervical lesions and cervical DH are positively correlated. This observation was connected to the pulp's close proximity to the bottom of deeper lesions and the number of exposed dentinal tubules, both of which may cause a painful response [11, 48]. According to reports, erosion has emerged as a significant risk factor for DH in recent years. First, erosion creates a tooth surface free of plaque, then it dissolves the tooth's outer layers, causing irreparable loss of hard tissue. This development may lead to DH because it allows dentinal tubules to open through the pulp to the oral environment [2, 49].

The present research demonstrated a strong association between bruxism and DH, which is congruent with prior findings [2, 17, 25, 50]. It is found that parafunctional behaviors and bruxism are plausible risk factors for noncarious lesions [51]. Because to the fact that the force magnitudes of bruxism are much greater than those of normal functional activity loads, occlusal parafunction is more likely than other parafunctional behaviors to result in dental tissue loss in the cervical region [11]. Moreover, the higher incidence of DH among bruxism sufferers in the current research might be explained by the fact that DH is often linked to noncarious cervical lesions [17].

Dentin sensitivity rises with age, according to two studies including people aged 12–20 and 18–35 [3, 52]. Several studies found a greater incidence rate in the ages 36–45, 40–49, and 50–59 [53–55]. Alcantra et al. discovered a weak statistically negative association between age and DH, which corresponds to recent findings [2]. Variations in the age-dependent distribution of DH may account for the disparity between the study populations. Age-related differences in the dentin-pulp complex may explain the study's negative association. Dentin sclerosis, secondary and tertiary dentin development, permeability, and hydraulic conductivity may all contribute to a lower risk of developing DH [56, 57].

Some investigations indicated that a cold stimulation might induce DH [4, 23, 35]. Several investigations have shown that acidic substances such as soft drinks may induce DH [7, 12, 15]. It was recognized that carbonated soft drinks induce tooth wear by eroding the enamel surface, followed by the development of DH [15]. Similar to earlier research, cold air, cold drinks, and sweet nutrition produced DH in the present investigation [47, 58]. This variance in the outcomes of the research may be due to the different food patterns of the populations [12]. Similar to the present research, other investigations have shown a favorable relationship between gingival recession and dentin hypersensitivity [10, 59, 60]. Since the thin layer of supra-dentinal cement is removed quickly, it may be stated that root exposures increase the susceptibility of the tooth tissue to the impression of hypersensitivity [11, 20, 61]. It has been found that dentin sensitivity rises proportionally with the number of remaining teeth in a person. It was determined that this discovery may be connected to the existence of oral hygiene practices that expose dentin tissue on the cervical surface of teeth [18]. According to Alcantara et al., a healthy tooth is a factor that protects against DH. This discovery was linked to the hydrodynamic theory [62]. The DH occurs in teeth when the dentin is exposed to the oral cavity via open dentinal tubules that provide a direct link between the oral environment and the tissue's internal pulp [63]. The interaction of exposed dentin with external stimuli might therefore cause pain in the teeth [19]. The postulated defensive capacity of a healthy tooth is based on the integrity of the enamel, which may restrict the intratubular fluid from being stimulated by external substances. In this situation, the DH in the healthy tooth may be linked to a defect in the cementoenamel junction that results in the exposure of dentine tissue [42]. In accordance with the literature, we identified a negative correlation between DH and the number of missing teeth index (MT), as well as the DMFT index.

There were several stimulation and assessment approaches for DH, and a gold standard method has not yet been developed [64]. According to Holland et al., the stimuli for measuring DH can be thermal, evaporative, or tactile, with the weakest stimulation administered first [65]. There are several tests for evaluating DH that are useful in triggering DH [66]. It was believed that the cold water test had a high level of specificity, or the ability to rule out hypersensitivity with a low rate of false positive findings [40]. In the literature, it was advised that at least two test techniques be utilized to diagnose DH [67]. Barose et al. used a clinical testing method with a clinical survey questionnaire that made it possible to exclude teeth with prosthetic preparations, cavities, restorations, and pulp alterations, which can cause pain and confound the diagnostic, in their study to reduce the possibility of bias, provide more reliable results, and enhance the internal validity in assessing DH [40, 68]. In the present investigation, thermal and evaporative tests, together with a clinical questionnaire, were employed to evaluate DH.

In the present study, while the extent of gingival recession was quantified, the loss of attachment in the gingiva was not assessed. It has been suggested that focusing solely on gingival recession may not sufficiently elucidate the relationship between DH and periodontal health. The results could potentially be skewed if gingival recession is the sole index examined, particularly in individuals who only exhibit a loss of attachment [34]. It might be beneficial to consider more comprehensive data, such as gingival attachment and the presence or absence of periodontal disease, to better understand the predictive factors of DH.

The lack of examination of the soft tissue phenotype represents a limitation of the study, as it is a critical factor in understanding individual variations in assessing and treating dentine hypersensitivity. This oversight may have resulted in an incomplete understanding of the factors contributing to dentine hypersensitivity. Future research should consider incorporating an analysis of the soft tissue phenotype to provide a more comprehensive and nuanced understanding of dentine hypersensitivity.

The cross-sectional design of the studies has been identified as a potential limitation. However, cross-sectional studies are considered valuable for identifying risk factors to be further explored as definitive risk factors in subsequent longitudinal evaluations [3]. It is also crucial to conduct DH assessments in future studies at varying time intervals, taking into account potential lifestyle differences that may influence the condition [1]. It can be inferred that early diagnosis of DH or the identification of predisposing factors enhances the likelihood of managing these factors, thereby reducing the incidence and prevalence of DH in adults [3].

Even though the study's inclusion of a subset of the population attending the university clinic in Turkey may restrict the generalizability of the findings, the frequency of self-reported hypersensitivity in adults in Turkey was 32.8%, and clinical examination of teeth revealed a prevalence of 14.57% for thermal DH and 3.90% for evaporative DH. Significant risk factors for both thermal and evaporative DH include cold air, consumption of sweet food, the presence of noncarious cervical lesions, and gingival recession. In the current investigation, the cold stimulus induces more sensitivity than the evaporative stimulation. It can be concluded that to fully identify the risk variables and implement the most effective preventative measures, further epidemiological research in this area is still required.

## Data Availability

The datasets used for the current study can be made available from the corresponding author upon reasonable request.
